# Influence of confinement on free radical chemistry in layered nanostructures

**DOI:** 10.1038/s41598-019-52662-z

**Published:** 2019-11-20

**Authors:** Khashayar Ghandi, Cody Landry, Tait Du, Maxime Lainé, Andres Saul, Sophie Le Caër

**Affiliations:** 1 University of Guelph, Department of chemistry, Guelph, ON N1G 2W1 Canada; 2 Université de Sherbrooke, Faculté de médecine, Sherbrooke, QC J1H 5N4 Canada; 3 LIONS, NIMBE, CEA, CNRS, Université Paris Saclay, CEA Saclay, F-91191 Gif-sur-Yvette, Cedex France; 4 Aix-Marseille University, CINaM-CNRS UMR 7325 Campus de Luminy, F-13288 Marseille, Cedex 9 France

**Keywords:** Solid Earth sciences, Chemistry, Energy science and technology, Engineering, Materials science, Mathematics and computing, Nanoscience and technology, Physics

## Abstract

The purpose of the present work was to study how chemical reactions and the electronic structure of atoms are affected by confinement at the sub-nanometer scale. To reach this goal, we studied the H atom in talc, a layered clay mineral. Talc is a highly 2D-confining material with the width of its interlayer space close to angstrom. We investigated talc with a particle accelerator-based spectroscopic method that uses elementary particles. This technique generates an exotic atom, muonium (Mu), which can be considered as an isotope of the H atom. Moreover, the technique allows us to probe a single atom (H atom) at any time and explore the effects of the layered clay on a single ion (proton) or atom. The cation/electron recombination happens in two time windows: one faster than a nanosecond and the other one at longer than microseconds. This result suggests that two types of electron transfer processes take place in these clay minerals. Calculations demonstrated that the interlayer space acts as a catalytic surface and is the primary location of cation/electron recombination in talc. Moreover, the studies of the temperature dependence of Mu decay rates, due to the formation of the surrogate of H_2_, is suggestive of an “H_2_” formation reaction that is thermally activated above 25 K, but governed by quantum diffusion below 25 K. The experimental and computational studies of the hyperfine coupling constant of Mu suggest that it is formed in the interlayer space of talc and that its electronic structure is extremely changed due to confinement. All these results imply that the chemistry could be strongly affected by confinement in the interlayer space of clays.

## Introduction

Confinement within nanostructures can affect the chemistry of atoms and molecules^1–13^. The more confined the nanostructure, the larger the expected effect of the confinement on electronic structure and chemical dynamics^6,14–17^. The kinetics of elementary reactions is the best probe of the effects of confinement on chemical dynamics; the most sensitive probe of confinement effects on electronic structure is the one that measures the electron density. The isotropic hyperfine coupling constant (HFCC) is very efficient in this regard^15–20^. It measures the strength of the coupling between unpaired electrons with the nuclear magnetic moment. HFCC is proportional to the electron spin density at the nuclei^14–26^.

In this work, we use HFCC to characterize free radicals. After its characterization, the electron transfer and reaction dynamics, which lead to free radical formation or decay, are reported in a confined environment. We also determine how the electronic structure changes under sub-nanometer confinement, which is a particularly interesting scale. In particular, we address some unanswered questions related to confinement at its smallest level: How are chemical reactions affected by the combination of surface and confinement effects at the angstrom scale? How does the electronic structure change under angstrom-scale confinement? What is the effect of extreme confinement on the reactivity induced by ionizing radiation?

For this purpose, a good starting point is to study the effects of confinement on the H atom, which is the most fundamental entity in chemistry. Indeed, investigation of H atom and its isotopes has played a fundamental role in the evolution of modern science^14–20,27–32^; as such, determining the behavior of H atom in various environments is crucial in the development of dimensionally constrained systems such as heterogeneous catalysts^31^, nanometer-scale semiconductors^32^, and hydrogen storage devices^29^.

Clay is an excellent medium to study the effect of confinement on chemical reaction channels and on the electronic properties of the H atom. Indeed, it provides natural abundant two-dimensional *layered* nano- and sub-nano- structures^33^. They are able to confine molecules and have the potential to provide catalytic surfaces at the same time^33^. The structure of clays also leads to a large surface area, as well as swelling, and ion exchangeability properties^34^. Finally, the compositional and structural features of layered clay minerals enable them to be modified by a large variety of polymers, organic and biological molecules^35^.

**Figure 1:**
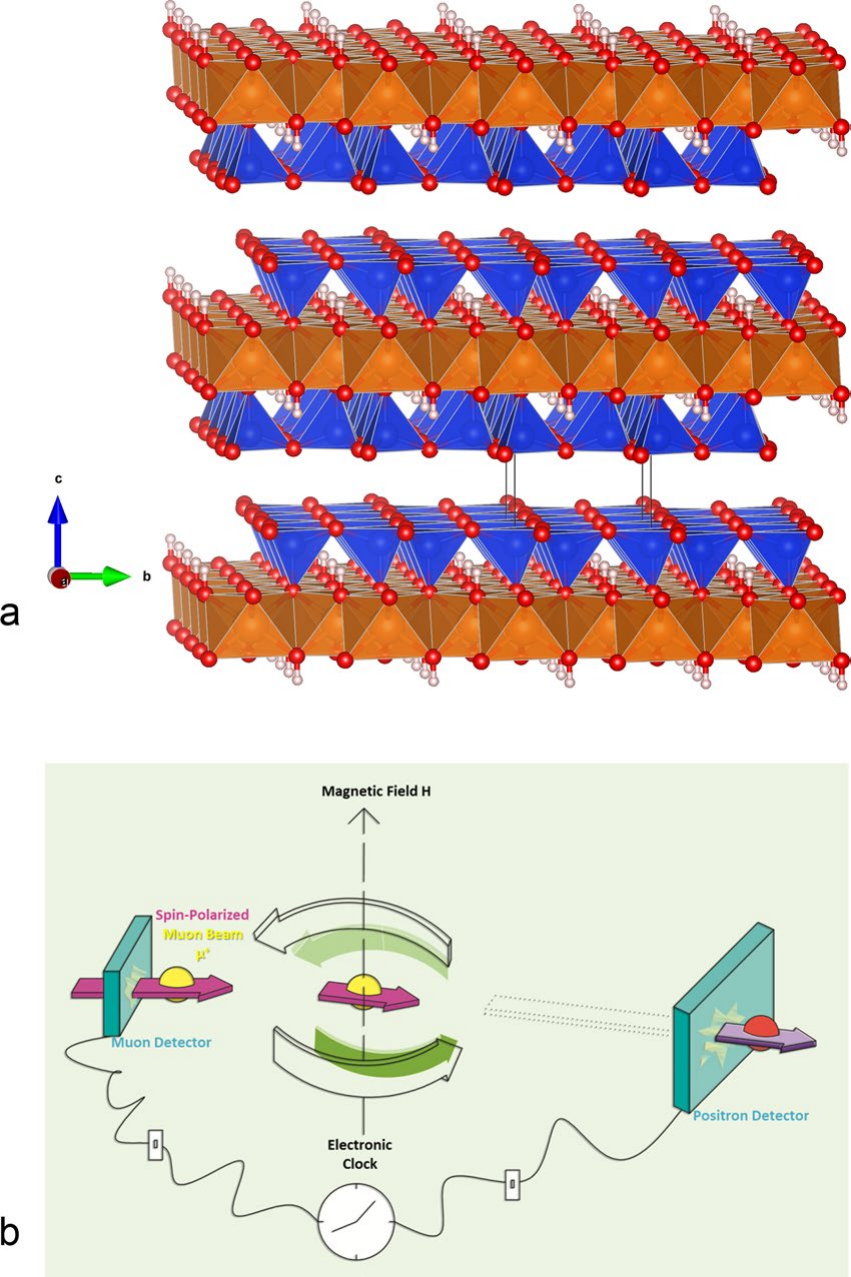
(**a**) Layered structure of talc (figure obtained with the VESTA software)^37^. An octahedral sheet (O) with magnesium atoms is sandwiched between two tetrahedral sheets (T) containing silicon atoms. Each sheet has a thickness of about 2 Å^89^. The interlayer space between TOT layers is about 3 Å. Mg: orange; Si: blue, O: red; H atoms: light pink. (**b**) Schematic diagram of μSR. See the methods section for description.

We will focus here on talc (Si_4_O_10_Mg_3_(OH)_2_). It is a phyllosilicate with a layered structure (Fig. 1a)^36,37^. One octahedral sheet (O) with magnesium atoms is sandwiched between two tetrahedral (T) sheets of silica. Each TOT layer is separated by an interlayer space around 3 Å wide (although considering the van der Waals radius of the atoms on the surface of the interlayer this space is close to 1 Å). This empty space^36^ provides a confined environment to do chemistry. Moreover, as commercial materials, talc and clays play important roles in various applications such as catalysts in chemical industry^38^ and waste management in the nuclear industry^36,39^.

There are two types of surfaces in talc (basal and edges). Basal ones are the surfaces limiting the interlayer space and the most abundant ones, while the edges, created by the breakage of the Si–O or Mg–O bonds, are the other surfaces not in the interlayer space^40^. These two types of surfaces exhibit different behaviors towards molecules. Basal surfaces are hydrophobic whereas edge surfaces are hydrophilic^40^. The surface chemistry of talc and other phyllosilicate minerals have been investigated by titration, adsorption and electrophoretic measurements^41^. Based on the surface chemistry of edges in talc it was suggested that they induced certain catalytic activities^42^ such as peptide bond formation by activating reactants^43^. We report here catalytic behaviors of the basal surfaces (towards electron transfer reactions and hydrogen formation as described later).

Electron transfer following irradiation can be used to generate H atoms or their isotopes in materials^18,19,36,44^. This has indeed been shown for talc^36^. The reaction e^−^ + MgOH → MgO^−^ + H is an electron transfer reaction that was reported in talc^36^. This reaction followed by dimerization of H atoms accounts for the H_2_ production in talc under irradiation^36^. Studying electron transfer reaction dynamics, H atom chemical dynamics and determining the electronic structures of H atom in clays, over a wide temperature range is a demanding task. It requires a technique that: i) can be used at any temperature; ii) is not limited by optical detection (as it is impossible for clays); iii) is time-resolved to be able to detect short-lived species; and iv) can provide the electronic structure of the free radicals. The only way to fulfill all above criteria is to use the hydrogen surrogate, muonium, and positive muon-based spectroscopic techniques as the positive muon (µ^+^) (called hereafter muon) is an exotic particle behaving as a light proton (H^+^)^19,20,44^.

Muonium (Mu ≡ μ^+^e^−^), obtained after capture of an electron by a muon, has one-ninth of the mass of H with almost the same Bohr radius (0.53 Å) and ionization energy (13.539 eV for Mu and 13.598 eV for H)^20^. Because Mu is electronically equivalent to an H atom, it can be used as a surrogate of the latter when it cannot be studied directly^45^. Examples are H atom in ionic liquids^19^, in ionic solids^20,28^ and in semiconductors^46,47^. This latter case is in particular important for talc, since the band gap of talc is calculated to be 5.3 eV, proving that it is a semiconductor^48^. Since Mu can serve as a surrogate of H atom^49–52^, it can also be added to molecules to form free radicals^18,25^. Among many works on use of Mu as surrogate of H atom, those that measured the isotropic HFCC are most relevant to our work in this paper. For Mu in most solids, such as diamond^50^, C_60_ fullerenes (and K_4_C_60_ and K_6_C_60_)^51,53^ and sulfur^52^, the lowest energy site is in the center of “cages” made by surrounding nuclei in the lattice. Also in all above-mentioned studies of Mu in solids and fluids, Mu HFCC is smaller than the Mu HFCC in vacuum with smallest values in Si and Ge (other than shallow donor semiconductors). When Mu is in a large enough cage, (e.g. in C_60_ where it has ~0.71 nm diameter) the decrease in HFCC is rather small due to weak Van der Waals interactions between Mu and the cage^51,53^. However in smaller three-dimensional cages in semiconductors (like in Si and Ge) the HFCCs are much smaller (close to half the vacuum value)^44,46,47,49,50^. Also in all above-mentioned studies of Mu in solids or fluids, HFCC mostly decreases with temperature. The calculated HFCC in sulfur is in particular interesting^52^. While Mu HFCC in the center of the cage (energetically preferred site) is 4013 MHz (which is slightly smaller than the value in vacuum), changing the distance from the center by 50 pm strongly affected the HFCC value (4013 MHz versus +50 pm/1654 MHz and −50 pm/2339 MHz) but did not seem to significantly affect the energetics. This suggests there should be a very large negative temperature dependence of HFCC. The distance between the sulfur atoms is 400 pm in their model which is larger than interstitial distances in Si and Ge but smaller than the space in C60.

## Results and Discussions

**Figure 2:**
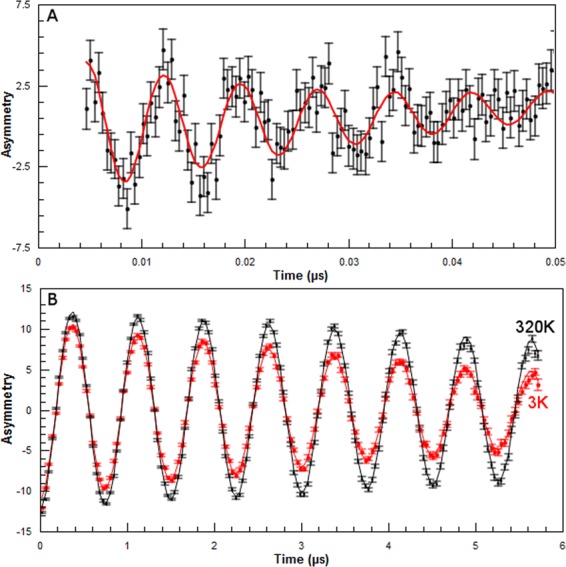
Time-domain asymmetry, expressed in percentage, in synthetic talc at (nominally) 100 G. (**A**) Decay rate of Mu. Data recorded at 3 K within a 0.05 μs time range. The black dots and bars represent experimental data points and uncertainty, respectively, whereas the red line indicates a theoretical fit to the data using Eq. 1 reported in Methods section. (**B**) Decay rates of the positive muon (µ^+^). The data were recorded at 3 and 320 K within a 6 μs time range. The black dots (resp. red) and line represent the experimental data and theoretical fit (to Eq. 1 in Methods section) at 320 K (resp. 3 K).

**Figure 3:**
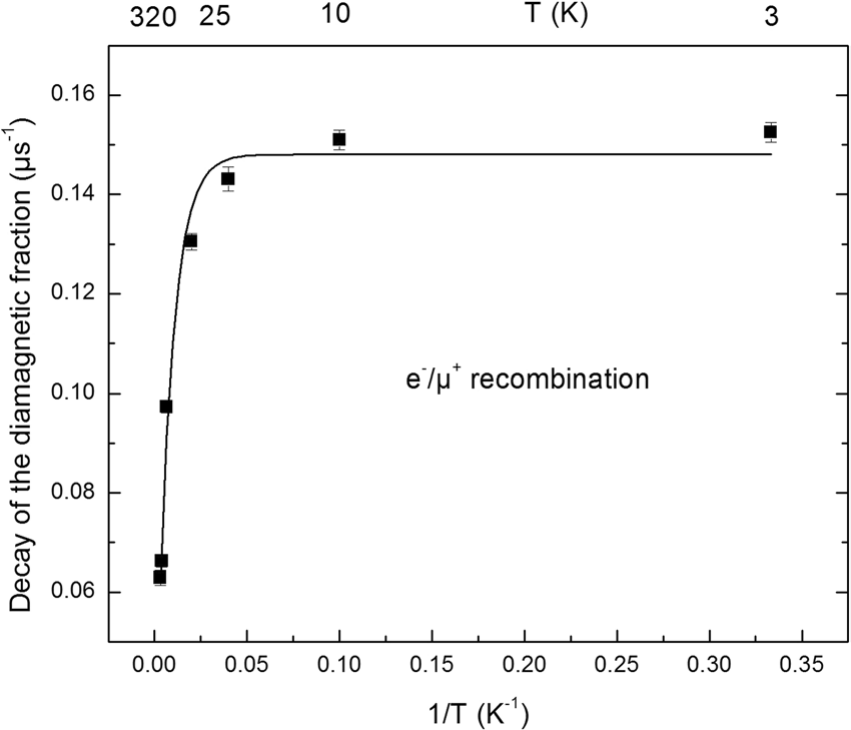
The decay rates of the diamagnetic fraction as a function of T^−1^. T is the temperature. The error bars are statistical uncertainties. The line corresponds to an exponential fit (see text).

Talc synthesis and characterization are detailed in the Methods section and in the Supplementary Information (Figs 1–3). The unit cell parameter along the c axis, *d*_001_ (measured by X Ray Diffraction), for talc is 9.43 ± 0.02 Å (see Fig. 1a) showing that there is no water layer in the interlayer space^36^. The thickness of TOT is 6.4 Å (Fig. 1a).

The principle of the muon-based technique used in this work is described in the Methods section and illustrated in Fig. 1b. The reactions we probe are electron transfer and H atom reactions by using Mu formation, muon spin relaxation^54,55^, and Mu decay^56,57^. Investigating Mu remains one of the most effective ways of providing information in solid samples on the dynamics and electronic structure of hydrogen atoms^19,20,28,56,58–61^. The knowledge of the electronic structure can be obtained via the determination of the HFCC of the trapped Mu (H) atom.

The HFCCs and the yield of H atom or free radicals were obtained following the muon spin precession in a transverse magnetic field (Figs 1b and 2). This is similar to free induction decays in NMR and pulsed ESR. If the positive muon does not couple to an unpaired electron, the spin precession is at Larmor frequency (Fig. 2B). This is the case for free muons (like H^+^)^62^ and also if it binds to a lone pair (like the lone pairs on oxygen in diamagnetic molecules, e.g. H_2_Oµ^+^ ~ H_3_O^+^). The fraction of these muons represents the diamagnetic fraction. The oscillation of spin polarization of muons coupled to an unpaired electron leads to a faster spin precession^18,25^, depending on HFCC (Fig. 2A). In talc, this oscillation decays in roughly 0.05 µs, showing that the species (confined “H” atom in the interlayer as explained later) is extremely reactive.

The information on chemical dynamics is obtained in two ways; one is by following the sub-ns thermalization process that leads to the formation of different species, such as Mu and diamagnetic species^19,20,54,63^. This is obtained by the analysis of the amplitudes of time-dependent spectra of the different fractions (Fig. 2). The other one is by studying the decay rates (e.g., Mu in Fig. 2A and diamagnetic species in Fig. 2B). To measure the decay rates we fitted the time domain spectra, like in Fig. 2, to exponential decays (the best fits were exponential functions).

### Decay rate of the diamagnetic fraction

The decay rates of the diamagnetic fraction as a function of temperature in talc from time domain fits of the spectra over roughly 6 µs are displayed in Fig. 3. The temperature dependence of the decay rate of the diamagnetic fraction is fitted well by the following expression: 1/τ = (0.148 ± 0.001) + (−0.127 ± 0.004) exp(−T_0_/T) with T_0_ = 122 ± 8 K and τ is in µs. As can be seen from the small error bars, the uncertainty on the relaxation rates is very small. Figures 2B and 3 clearly indicate that the decay rate decreases when temperature increases. For temperatures below 25 K, the decay rate is constant.

The observed decay rates are due to rate of electron transfer to muons (like electron transfer to H^+^) at the microsecond time scale. The decay rates suggest that the diamagnetic fraction is mostly due to positive muons or to positive muons bound to oxygen lone pairs. Indeed, a molecular species like MuOH would have a slower reaction rate^23^. The spin relaxation of positive muons in a diamagnetic solid material is typically due to dipolar interactions between positive muons and dipoles. This could not be a potential mechanism for the observed decay rates, because such relaxation rates are usually an order of magnitude smaller than what is observed here^64–67^. Furthermore, the only spin-active nuclei with significant abundance in synthetic talc are protons, which are in MgOH sites in the layers (Fig. 1a). They are less abundant than other nuclei in talc.

The fact that we observe this electron/cation (muon) recombination (or interaction due to polaron generation) rates at the microsecond time scale suggests the existence of electron transfer processes with much slower rate than the ultrafast electron transfer leading to Mu formation, which is more than four orders of magnitude faster (faster than ~10^10^ s^−1^)^19,20,23,54^.

Electron transfer processes in clay minerals are important from an applied point of view, since clays can serve as host materials for photochemical reactions^68^. They can also be used for the electron-transfer based polymerization of some organic compounds^69^ and are known to catalyze redox reactions^70^. In addition, the radiation damage to a material is in part due to electrons that are formed from ionization of the material. The study of the interaction of ionizing radiation in clays has important applications as clays are used as a natural barrier in the deep geological disposal of high-activity and medium-activity long-life nuclear waste^71,72^. Only a few studies have up to now dealt with this important topic^36,72^. Also, due to the solid form of the samples, no kinetic data were reported so far^72^. Our observations here imply that there are two types of radiation damage and electron transfer reaction in clays. The first one is fast and occurs within less than 0.1 ns and the other is much slower and happens within microseconds (and longer).

This implies that there are two types of electrons in the layered nanostructure of talc. Note that this is formed here by radiation but this can be extended more generally to all cases in chemistry or material technologies where free electrons are formed. The ultrafast electron transfer that has been observed causing Mu formation (see the signal in Fig. 2) is most probably due to the electron gas behavior that is free in the interlayer space. The interlayer space provides a free space in two-dimension for hot electrons (electrons with energy larger than thermal energy). This is consistent with the results at all temperatures, and even at very low temperatures (close to 1 K) where the electron mobility is more than four orders of magnitude larger than in the slow electron transfer reported in Fig. 3. If the electrons participating in the ultrafast process were not in a free electron gas state, we would have expected a very significant drop of the amplitude of Mu at the lowest temperatures we studied. This also suggests that both cations (positive muons) and “free electrons” are confined in the interlayer space (see Fig. 1a).

### Cation implantation

**Figure 4:**
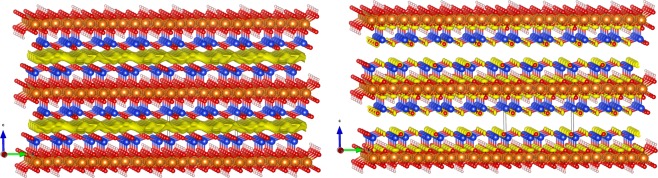
(**a**) Attractive electrostatic potential exerted on the muon calculated at −0.83 Ry (left figure). The most favorable space for the cation is the interlayer space (yellow color). (**b**) Total electron density near the Fermi level (right figure).

To test the above hypothesis and to understand the inverse Arrhenius temperature dependence of the cation (muon)/electron recombination rate, the location of the cation (muon) must be understood. Calculations were performed in order to follow μ^+^ implantation as a probe for cation sites within talc. The details of calculations are explained in the methods section. The results are displayed in Fig. 4a. The cation is implanted preferentially in the regions with a large negative electrostatic potential (which corresponds to large positive values for electrons). Moreover, electrons are more likely to be extracted from the system in regions where the electron density is close to the electrochemical potential for electrons (Fermi level).

In our calculations, the sum of the ionic and Hartree potentials for the electrons ranges from large negative values close to the nuclei, to a maximum of 0.87 Ry. From Fig. 4a displaying the attractive potential for the point positive charge (positive muons in this case) calculated at −0.83 Ry, it is clear that the middle of the interlayer space is the most favorable location for muons (protons, or in general cations) as shown by the yellow region. This is an electrostatic confinement to almost a two-dimensional plane, which confirms our hypothesis that the interlayer space is the favorite location for positive charges in clays. It can also have significant implications for both the catalytic properties of talc for electron transfer reactions and for its semiconducting behavior (in particular the location of positive charge carriers in semiconductor clays) suggesting that exchangeable catalytic cations could be located in this space. We believe that these features enable clay minerals such as talc to serve as a class of excellent supports for immobilizing catalytic cations.

Figure 4b presents the total electron density near the electrochemical potential of electrons. This shows the sites that are the most prone to ionization. It is clear that these sites are located close to all oxygen atoms. Among these oxygen atoms, there are those in contact with the interlayer space, and hence, near the cations.

Muons are preferentially thermalized in the center of the interlayer space. Electrons released from ionization sites near this space begin to move towards these muons. During this process, when the electrons are within the range of the muons/cations they create neutral species (Mu). Note that the released electrons have a greater energy than the trapped electrons, so their wavefunction will be delocalized, making the electron capture more favorable. This should however be at energies close to thermal energy^73^. The electrons that are not close enough and do not form Mu can cause the relaxation of the diamagnetic signal described above (Fig. 3). They are farther and therefore react with positive muons at the microsecond time scale. Hence, the capture rate of these “slow” electrons will be the rate of the diamagnetic muon decay.

Clearly, this capture rate has a non-Arrhenius and indeed an inverse Arrhenius behavior. This could be due to following reasons: (i) there would be more slow electrons in the interlayer at lower temperatures, due to more radiation-induced ionization at lower temperatures; (ii) two different electron transfer reactions could compete, i.e. electron capture by the muon and by other species (within the layers), such as MgOH groups (the decay rate being equal to *k [e*^−^], *k* being the rate constant and *[e*^−^] the electron concentration); (iii) the muon/electron capture rate increases when the relative kinetic energy of them is lowered as shown by recent theoretical quantum electrodynamics predictions^73^; (iv) the electron capture is more favorable when the lattice is closer to the equilibrium structure. At higher temperatures, many lattice degrees of freedom are excited and this could lead to an unfavorable condition for electron capture.

We will show below (see Fig. 6 and the related discussion) that only (i), (iii) and (iv) can be the main mechanisms to account for our observations. We can rule mechanism (ii) out, since such a mechanism would cause a non-exponential muon decay that we did not observe (see e.g. Fig. 2b).

### Mu decay rate

**Figure 5:**
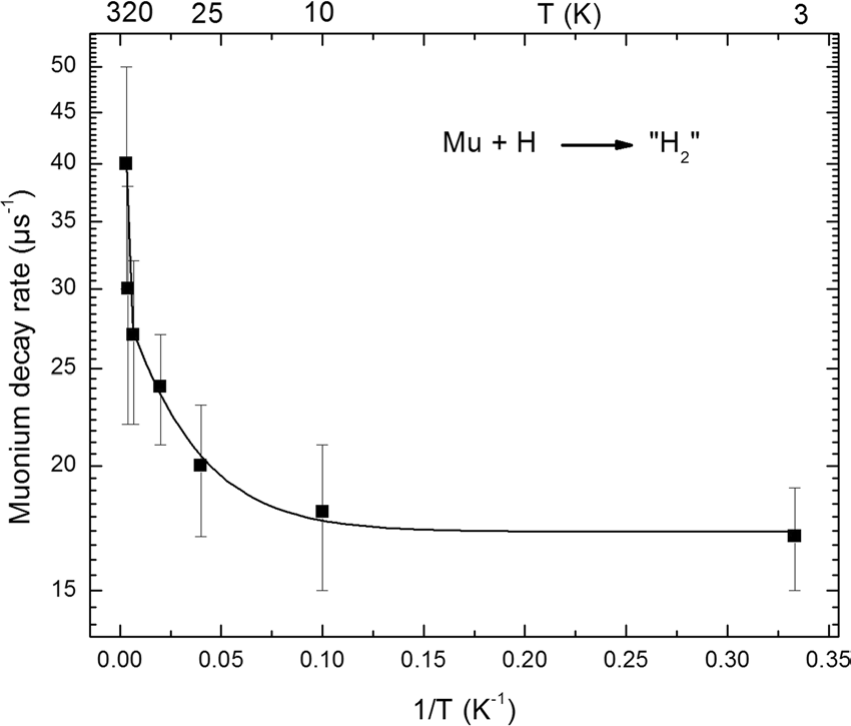
Arrhenius plot of Mu decay rate. The line is an exponential line corresponding to the best fit of the data.

Figure 5 displays the Arrhenius plot of the Mu decay rates. In contrast with the latter case (Fig. 3), this decay rate increases when temperature increases. The following expression fits best to experimental data: 1/τ = 17.18 + 5737 exp (−T_1_/T) + 12.4 exp (−T_2_/T) with T_1_ = 1984 K and T_2_ = 33 K. 1/τ is expressed in µs^−1^ and the temperatures (T) is in K.

The temperature-dependent decay is, in principle, due to one of the following three mechanisms. (i) The Mu atom encounter electrons in the clay. The spin exchange^19^ between these electrons and the electron in Mu leads to spin relaxation. The addition reaction (of electrons to H (Mu)) could, in principle, lead to hydride ion (H^−^) formation as well^58^. (ii) The reaction of Mu with holes in talc. Studies of ionizing radiation of talc have proven that the radiation-induced holes are located on oxygen atoms (linked to magnesium or silicon atoms), as shown by ESR experiments^36^. (iii) The Mu + H reaction forming MuH, which is equivalent to the H_2_ formation under ionizing irradiation^36^.

This latter mechanism is the most likely one for the following reasons: i) holes are not mobile, yet they have the same concentration as electrons, and electrons and H usually outcompete holes to participate in reactions with Mu; ii) a previous work on the interaction of ionizing radiation with talc has shown a large concentration of H_2_ that was not accounted for by stable H atom concentration^36^. This has been associated with an unobservable H + H reaction in talc^36^. Furthermore, pulse radiolysis experiments performed in nanoporous silica have attributed most of the decay of solvated electron in the ns-µs timescale range to the e^−^ + SiOH → SiO^−^ + H reaction^74^. This suggests that the reaction with Mu, which is happening in the same timescale, occurs with H atoms and not with electrons, as electrons will react preferentially with MgOH groups in the present case.

It is known that H atoms are generated by radiolysis of MgOH groups in the layers^36^. Therefore, the decay rates we report here are due to diffusion of H and Mu towards each other to form MuH. Our results show that there is a diffusional barrier around 18 kJ.mol^−1^, but at low temperatures, the transport takes place via quantum diffusion with an activation barrier of 0.3 kJ mol^−1^. It is expected that the temperature where quantum diffusion starts would be lower for H diffusion due to its larger mass as compared to Mu. This quantum tunneling occurs for a distance close to 1 to 3 Å (between Mu close to the center of interlayer space and H close to MgO). Our results therefore suggest that the relaxation mechanism of the muon in Mu is due to Mu and H diffusion (which is thermally-activated above 25 K and is due to quantum diffusion below 25 K). This implies that the clay material could be considered as a quantum material at low temperatures, with a much larger temperature expected for electron quantum transport and a lower temperature expected for hydrogen atom quantum transport. This is to our knowledge the first report of chemical dynamics in the interlayer space of clays, and the first report of quantum transport and quantum effects on chemical reactions in talc.

### Temperature dependence of different fractions

**Figure 6:**
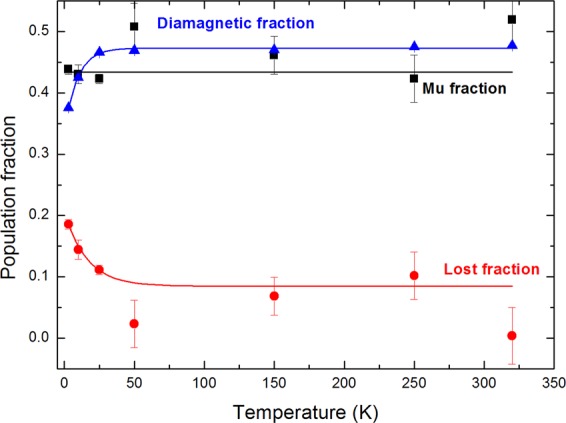
Evolution of different population fractions (diamagnetic fraction, Mu fraction and lost fraction) as a function of temperature. The lines are shown to help the reader locate the data points for each fraction.

The temperature dependence of different fractions of muoniated species (diamagnetic fraction, P_D_; Mu fraction, P_Mu_ and lost fraction^19,20,23^ which is P_L_ = 1 − P_Mu_ − P_D_) is displayed in Fig. 6.

The Mu fraction is less sensitive to temperature. Indeed, Mu is formed via ultra-fast electron transfer to positive muons^19,20^:

$${{\rm{\mu }}}^{+}+{{\rm{e}}}^{-}\to {\rm{Mu}}$$

The diamagnetic fraction increases in the 0–50 K temperature range and then remains constant. The lost fraction is due to the reactions of electrons and H atoms in the radiation track within clays with Mu in 0.1 to 10 ns. The lost fraction decreases with temperature. Considering that the trend depicted in Fig. 3 is similar to the trend shown for the diamagnetic fraction (but in opposite directions *vs* temperature), it is possible that the increase in the diamagnetic fraction with temperature is linked to the fact that this population decays slower when temperature increases, and with an almost constant rate for temperatures below 50 K.

This similar temperature dependence could be a coincidence, considering that the two mechanisms involved have more than three orders of magnitude difference in time scale. Considering however that one potential mechanism behind the temperature dependence shown in Fig. 3 is the increase of radiation-induced damage (increase in electron concentration and hence Mu (H) atoms) with the decrease in temperature, this link seems to be non-coincidental. In this case, we expect more lost (or Mu) fractions, as they are mostly due to electrons formed by radiation damage, and a smaller diamagnetic fraction at lower temperatures. Certainly, the diamagnetic and lost fractions show this trend. Therefore, we conclude here that radiation damage increases when temperature decreases.

### HFCC coupling constant

The measured HFCC (in this section) is used with our computational data to further understand the nature of the observed Mu (H) atom and of its precursor in talc.

The following trends were observed (Methods, Fig. 2, and Supplementary Information Figs 4–9). We detect one type of Mu at all temperatures. This suggests that there is only one site for H atoms. The details are explained in the Methods section and also in the Supplementary Information. Although one way to interpret the experimental data is the observation of a 2D H atom due to large HFCC and its comparison with calculations^75–78^, the thickness of the interlayer space may be still too large to account for this interpretation. Therefore we will compare in the following our results with those obtained assuming a confined but three-dimensional space.

Note that the decay rate of Mu is very large at 320 K due to its reaction with H, making the measurement of the HFCC at this high temperature less reliable. Therefore, the HFCC value above 250 K is not presented.

**Figure 7:**
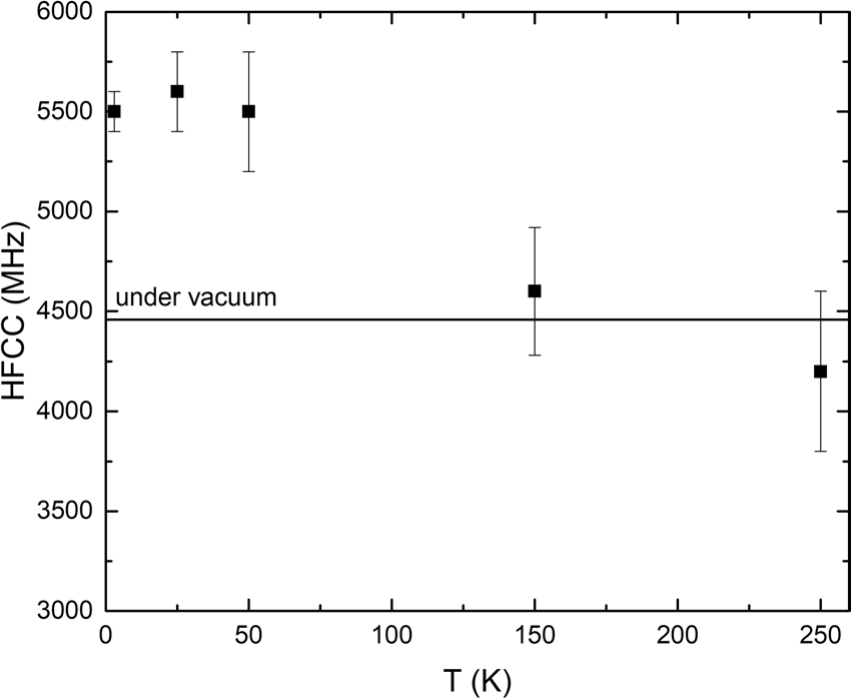
Evolution of the HFCC value of Mu with temperature. The line indicates the value measured in the vacuum.

The HFCC value of Mu globally decreases with temperature (Fig. 7). It ranges from 5500 MHz at 3 K to 4200 MHz at 250 K. The value at 3 K is ~23% larger than the value in vacuum (4459 MHz), while it is 6% lower at 250 K. The large value measured at 3 K is to our knowledge the largest HFCC value reported in any material. It is even larger than the one reported in stishovite (5170 MHz)^79^. Moreover, the large temperature decrease (almost 30% per 250 K) is to our knowledge the largest temperature dependence reported for Mu (H). Indeed, in water, the HFCC value increases with temperature by about 1% per 250 K^21^. The decrease we measure here is equivalent to a 30% decrease in electron density at the nucleus per 250 K. This corresponds to an electron density transfer to the atoms which are close in the clay mineral. Both the temperature dependence and the large value at 3 K are due to the highly confined environment of the Mu (H) atom in the interlayer space. This is also consistent with the observation of the kinetics data discussed above. Therefore, the highly confined environment affects both the electronic structure and the chemical dynamics, which is of crucial importance in the field of the catalytic applications of clays.

**Figure 8:**
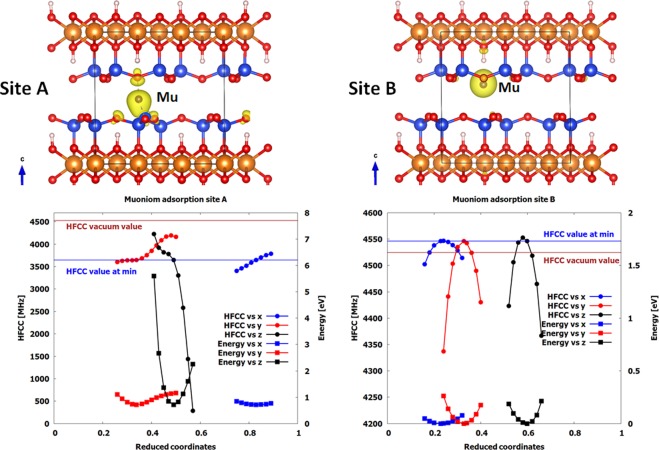
Left: One of the two potential final sites for Mu (H) atom (site A) in the center of the interlayer space. The energy and HFCC value are given as a function of displacement from the minimum energy position for site A. Right: One of the two potential final sites for the H atom (site B) close to the interlayer boundary. The energy and HFCC value are given as a function of displacement from the minimum energy position for site B. Top: Position A is in the middle of the interlayer space. Position B is in a hexagonal site close to the interlayer boundary. Position B has a lower energy than A by 0.7 eV. In all cases, oxygen, silicon and magnesium atoms are represented by red, blue and brown colors, respectively. The isosurfaces of the spin density at 0.007 eV/a_0_ are shown in yellow. Bottom: in all cases, the energies are plotted with squares and are visualized on the right y-axis. The HFCC values are plotted with circles and are visualized on the left y-axis. As references we include the HFCC value at the energy minimum (blue line) and the vacuum value calculated with the same program (brown line).

To understand the nature of the Mu (H) atom site and the negative temperature dependence of its HFCC value, calculations were undertaken at the same level as the electrostatic calculations described above. The corresponding results are displayed in Fig. 8.

The calculations suggest that two sites are possible for Mu (H). Position A (Fig. 8) is in the middle of the interlayer space while position B (Fig. 8) is close to the interlayer boundary. The lowest energy position is B and the energy difference between the sites is about 0.7 eV. The HFCC values of Mu at 0 K in sites A, B, and in vacuum are calculated to be 3639, 4543, and 4525 MHz, respectively. Therefore, the HFCC value in site A is smaller than that obtained in vacuum, whereas it is slightly higher in site B.

The reason for the smaller value in site A is the antibonding interaction between the muon and four oxygen atoms (one above and three below) which results in an effective decrease of the total spin density around the muon atom. A further effect that can explain the decrease of the HFCC value due to the decrease of the spin density at the muon site is the deformation of the spin density due to the electrostatic attraction of the positive Si atom below. This is similar to the observed Mu HFCC in Si and Ge^46,47,80^ while different with the cases where Mu was in a less confined space^51–53^. Concerning the larger HFCC value in site B than in vacuum, it is difficult to give a definitive answer. One probable explanation is that in this site the muon is on top of the –OH group, where there is almost no bonding with the neighboring atoms (see the isosurfaces of the spin density in Fig. 8). The first neighbors of this almost free Mu atom are six negative oxygen atoms, which repels the spin density increasing the spin density at the muon site. This should be the case as well for Mu in Stishovite^67^.

To check the energies of Mu at the two sites and the temperature dependence of the HFCC, the position of Mu was shifted along the three directions. The evolution of the energy and of the HFCC values as a function of the reduced coordinates (0 < x, y, z < 1) is displayed in Fig. 8 (bottom; left y-axis for HFCC values and right y-axis for energy). Clearly, any displacement increases the energy (Fig. 8), as shown by the parabolas with the positive concavity. Site A has an HFCC value that is smaller than that of vacuum. This value can increase by moving Mu in any of the three directions. As the energy increases very quickly (especially if Mu is moved along the z axis), the HFCC values are expected to increase with temperature. On the contrary, site B (corresponding to the lowest energy) has an HFCC value higher than the vacuum value. Furthermore, in this case, the maximum HFCC value is at the lowest energy point. One can then imagine that when increasing temperature the HFCC value should decrease, as the nuclear wave function with higher energy states will be populated. Based on the comparison of the experimental values of the HFCCs with the calculated values at 0 K and the predicted temperature dependencies of the two sites, we conclude then that site B corresponds to the location we observe experimentally.

**Figure 9:**
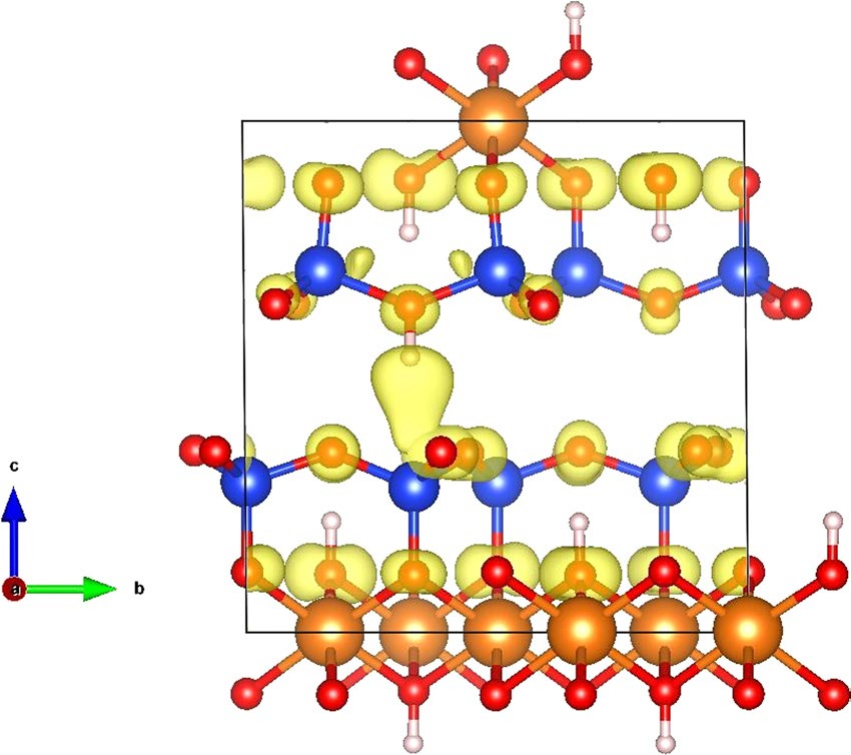
Calculated precursor having an HFCC of 185.3 MHz and being in a triplet state. The spin density is mainly located on the muon (in the interlayer space) and on oxygen atoms.

To understand the nature of the precursor of Mu, we analyzed the ratios of the amplitudes of the two frequencies associated with Mu at the magnetic field values used for each experiment. We conclude that the precursor of the species present in the interlayer space exhibits a low HFCC around 190 MHz (we call it species C hereafter). The transformation from C to Mu at site B occurs with a rate around 10^8^ s^−1^. With such a precursor and rate of Mu formation, the ratios of the amplitude of the higher frequency to the amplitude of the lower frequency (see Supplementary Information) decrease drastically when going from ~100 G to ~200 G, to the degree that the higher frequency signal would not be observed, and the amplitudes would be small in general^81,82^. Results from calculations have revealed some clues for this precursor (Fig. 9). It can only correspond to a Mu bound to a hole and being in a triplet state. The HFCC value of this species was calculated to be 185.3 MHz, in good agreement with the value determined experimentally. The spin density of C is localized at the muon (located in the interlayer space) and on oxygen atoms. This species will then form Mu in the interlayer space at site B via electron transfer from an electron to hole, and the subsequent release of Mu to site B.

## Conclusion

We have shown that a strong confinement (sub-nm), easily available in 2D-confining layered clay minerals such as talc, could have extreme effects on chemical reactions and on the electronic structure of free radicals. We have also evidenced that at least two types of electrons, with different characteristic time constants differing by at least three orders of magnitude, are generated in talc. This induces different radiation damage types in clays exposed to ionizing radiation, which is an important knowledge for the deep nuclear waste storage where clays constitute the geological barrier. Moreover, we have shown that the H_2_ formation mechanism is thermally activated above 25 K, while below 25 K it is due to quantum diffusion. This suggests that clay could be considered as a quantum material at low temperatures.

We have also evidenced that cations are preferentially located in regions with a large negative electrostatic potential, i.e. in the interlayer space. The reaction of cations with free electrons first leads to neutral chemical species located close to the middle of interlayer. This is however a local minimum on the potential energy surface. The atom (within less than 0.1 ns) then moves to the global minimum, which is close to the O atom at the border of the interlayer space (via electron transfer to the hole). This leads to the formation of a radical with a high HFCC value, in consistency with the existence of hydrogen in the interlayer space of talc. Due to extreme confinement, the HFCC value has a large temperature dependence. The interlayer space thus provides electrostatic confinement, which in turn induces large changes to the electronic structure of the atom (molecule) present.

Comparing our data in this work with previous studies, we discovered that the effects of confinement are multifaceted. One effect is on electronic structure of an atom or molecule in confined media. The effect of the confinement on the electronic structure of the Mu at sites A and B is reflected in the iso-surfaces of the spin densities (Fig. 8) and the HFCC^15–20^.

Site A is in the middle of the interlayer space and the electron transfer to the neighboring O atoms decreases its HFCC value (3639 MHz) significantly with respect to the vacuum value. Site B is close to the boundary of the interlayer space and the total spin density is almost entirely localized around the muon. The six neighboring O atoms repel the electronic density localizing further the spin density and increasing the HFCC value. This can be the reason for the large HFCC observed experimentally at lowest temperatures.

Another effect of confinements is on temperature dependence of the HFCC. Similar to the increase of HFCC, its large temperature dependency is an effect of confinement. This is similar to predictions for Mu inside a sulfur cage that was discussed in the introduction^52^. It is expected that the temperature dependence would also increase by making the potential energy surface and variation of HFCC with distance (Fig. 8) sharper in a more confined space.

The other effect of confinement is on the richness of chemistry, such as existence of different Mu sites (like having different catalytic sites in a catalyst nanostructure). Most probably, by decreasing the interlayer distance these sites would collapse into one site (one site would disappear). This will decrease the richness of chemistry inside the confined environment. Although in the first glance we also expect the reactions of electrons and protons and reactions of H and Mu (equivalent to H_2_ formation), become faster with decrease in interlayer space (increase in confinement) however with a drastic change to confinement we would also expect the changes to the atomic structures of the lattice. This means we would expect different effects that may cancel each other on the electronic structure and reaction rates. Moreover, to understand the kinetic controlled processes under confinement the barriers of different transformations should be calculated as a function of confinement. This certainly requires a long-term investigation of both theoretical and experimental effects of confinement to explore the multifaceted confinement effects.

Our results also give more insights into the properties of clay minerals as catalysts, and demonstrate the very particular role played by the interlayer space, which can act as a very interesting angstrom-scale catalytic reactor for electron transfer reactions. This has implications in the petroleum refining industry (catalytic cracking, hydrogenation and other processes), in organic synthesis and in environmental applications.

Confined layered systems such as clay minerals have then the potential to facilitate a rich chemistry, including the transformation of CO_2_ to value-added products. In addition, we showed that muon methods provide a powerful tool for the study of confinement effects and of reactivity under confinement. Their power lies in their ability to provide efficient and relevant probes of confinement in chemistry. These probes are parameters that are proportional to the electronic structure of free radicals on different nuclei and also to reaction rates of elementary chemical reactions at a wide range of temperatures, from ultra-low temperatures where quantum effects are manifested, to temperatures relevant for industrial applications. Studies such as the present one play a fundamental role in the evolution of dimensionally constrained interfaces, modern catalysis, semiconductors, electron transfer processes, quantum materials chemistry and energy technologies.

## Methods

### Talc synthesis

Synthetic talc was synthesized by hydrothermal synthesis from gels of appropriate compositions prepared according to the conventional gelling method described previously^83^. Characterization includes infrared spectroscopy (IR), thermogravimetric analysis (TGA), and x-ray diffraction (XRD) presented in Supplementary Information Figs 1–3.

### Characterization of talc samples

In order to record infrared (IR) spectra, 1% of sample was pelletized in KBr and then analyzed by a Bruker Tensor 27 FT-IR spectrophotometer. All the spectra were collected in transmission mode in the 4000–370 cm^−1^ energy range with a 4 cm^−1^ resolution from 100 scans. A pure KBr pellet was used for the reference.

Thermogravimetric Analysis (TGA) experiments were performed with a Mettler-Toledo TGA/DSC 1 analyzer. An alumina crucible of 70 µL containing approximately 20 mg of sample was heated at a heat flow of 10 °C.min^−1^ under a dinitrogen flux of 50 mL min^−1^ in order to reach a final temperature of 900 °C and then brought back to room temperature. Data were analyzed using the STAR^e^ software.

Powder X-ray patterns were recorded on a Bruker D8 Advance diffractometer equipped with a grazing parabolic Göbel mirror and a Cu emitter (λ_CuKα_ = 1.541 Å, 40 kV/40 mA). The diffracted beam was collected by a position sensitive Vantek detector.

### Muon spin spectroscopy

The spin polarization of the positive muons (called simply “muons” in this manuscript) is almost 100%. This and the preferential asymmetric emission of high energy positrons (that can be detected) along the direction of the muon spin at the time of decay (Fig. 1b) allowed us to probe the evolution of the muon spin, coupled (or not) to unpaired electrons^19,20^. The sample, talc, was implanted with positive muons (μ^+^) at the M20 muon channel of TRIUMF laboratory in Vancouver, Canada, and the muoniated products were studied in real time. Samples of talc were subjected to several degassing cycles to remove oxygen (pumping/flushing with nitrogen) and stored in an air-tight container for transportation to the facility. The samples were subsequently stored in an in-house made mobile glove box until the day of the measurements. They were then placed in an aluminized Mylar cell that was sealed with silver-foil tape, where it was subsequently transferred to a Horizontal Gas Flow (HGF) Cryostat (suitable for temperatures from 2.8 to 330 K) compatible with the LAMPF spectrometer. This spectrometer uses a Helmholtz coil which provides a maximum field of 4000 G and is suitable for transverse and longitudinal field studies. The muon beam arises from pion decay at rest and possesses ~100% spin-polarization. Thermalization begins as the muon interacts with the material and eventually reaches thermal equilibrium towards the end of the radiation track.

The spin of the μ^+^ is initially polarized opposite to the direction of its momentum (anti-parallel polarization). Before a muon interacts with the sample, it passes through a counter that sends an electronic signal to a time-digital-converter (TDC) and a counter is incremented, starting a “clock” (Fig. 1b). The positive muon after thermalization eventually decays to a positron and neutrino–antineutrino pair. The positron is emitted asymmetrically preferentially along the muon spin direction at the time of decay. When the positron is detected, the “clock” is stopped and the time intervals are collected in a histogram. Two positron detectors are positioned in the plane of the muon spin precession.

The asymmetries are fitted in time domain (and double checked for consistency with the frequencies from the Fast Fourier Transform (FFT) signals) using the following equation:

1 $$A(t)={\sum }_{i}{A}_{i}\exp (-{\lambda }_{i}t)\cos ({w}_{i}t+{\psi }_{i})$$

where, in a given environment, *A* represents the asymmetry of the muon fraction *i*, *λ*_*i*_ represents the muon relaxation rate, *t* is the time, *w*_*i*_ is the precession frequency, and ${\psi }_{i}$ is the initial phase of the given fraction. The smallest relaxation rate accessible by μSR is limited by the muon lifetime. The lowest relaxation rate that can be measured in a transverse magnetic field in a continuous muon beam source like the one at TRIUMF is close to 10^−2^ µs^−1^ ^44,64^.

Muons or muons incorporated in a diamagnetic molecule (e.g., MuH) precess at the muon Larmor frequency (13.554 kHz G^−1^). A fraction of muons capture electrons to form an atom considered as an isotope of the H atom, and which is called muonium (Mu ≡ μ^+^e^−^)^18,57,84^. Although muons in the form of Mu have four precession frequencies in transverse field, only two of these are low enough to be resolved by conventional detection apparatus:

2 $${\nu }_{12}=\frac{1}{2}[({\nu }_{e}-{\nu }_{\mu })-{({({\nu }_{e}+{\nu }_{\mu })}^{2}+{A}_{\mu }^{2})}^{\frac{1}{2}}+{A}_{\mu }]$$

3 $${\nu }_{23}=\frac{1}{2}[({\nu }_{e}-{\nu }_{\mu })+{({({\nu }_{e}+{\nu }_{\mu })}^{2}+{A}_{\mu }^{2})}^{\frac{1}{2}}-{A}_{\mu }]$$

where *v*_12_ and *v*_23_ are the resulting detected precession frequencies, *v*_*e*_ and *v*_*μ*_ are the electron (2.80247 MHz G^−1^) and muon (13.554 kHz G^−1^) Larmor frequencies respectively, and *A*_*μ*_ is the hyperfine coupling constant for muonium in its particular environment. Using this, we can determine the hyperfine coupling constant of muonium Mu in synthetic talc by finding the precession frequencies.

The spectra at TRIUMF, taken via the LAMPF spectrometer, were acquired by the use of a transverse magnetic field with respect to the muon spin. Bin size was set to 195.3125 ps and a total histogram length of 6 μs was used.

### Calculations

For the calculations, we used Quantum Espresso (QE)^85^ and the WIEN2K^86^ codes based on density functional theory. For the Quantum Espresso code we have used ultra-soft pseudopotentials and the Perdew, Burke and Ernzerhof (PBE) functional^87^ with a plane-wave and charge-density cutoff of 80 Ry and 320 Ry, respectively. We used a 9 × 5 × 5 Monkhorst-Pack^88^ grid for the first Brillouin zone sampling of the 42-atom triclinic unit cell. The code has been used to calculate the electronic density close to the Fermi level and the electrostatic potential exerted on the muon which is the sum of the bare ionic potential and of the self-consistent Hartree potential generated by the valence electrons (Fig. 4). The systematic search of the adsorption site for the Mu atom has been performed by including an additional H atom to the monoclinic cell. The calculations for the additional hydrogen assumed that the Mu atoms were spin-polarized. The hyperfine coupling constants reported in Fig. 8 and also for the charged cell in Fig. 9 have been calculated using the full potential linearized augmented plane wave plus local orbitals method as implemented in the WIEN2K code. The calculations were performed using the generalized gradient approximation of PBE for exchange and correlation and a cutoff parameter RK_max_ = 3. The radii of the muffin-tin spheres were set to 0.56 a.u. for H, 1.90 a.u. for Mg, 1.03 for O and 1.4 a.u. for Si. The energy difference between the A and B sites calculated with the QE code after full relaxation is 0.707 eV and 0.716 eV with the WIEN2K code. The hyperfine field calculated by the program (in kGauss) has been multiplied by *g*_*e*_ *g*_*μ*_*μ*_*μ*_/*2πℏ* to obtain the HFCC value in MHz at the muon sites reported in the manuscript. The value calculated for a free Mu atom, 4524 MHz, to be compared to be compared with the experimental hyperfine value of 4463 MHz. One can obtain a better agreement with the experimental value by correcting the spin density at the nucleus taking into account the difference in bohr radius between the H and Mu atoms. With this correction one gets 4458 MHz for the free Mu atom.

## Supplementary information

supplementary information
