# Supplementary material for: Influence of confinement on free radical chemistry in layered nanostructures
Source: Sci Rep. 2019 Nov 20;9:17165. doi: 10.1038/s41598-019-52662-z (PMC6868163; doi:10.1038/s41598-019-52662-z)
Supplement: Supplementary file 1 — supplementary information [file 41598_2019_52662_MOESM1_ESM.pdf]

Supplementary Information for
"Influence of confinement in layered nanostructures on free radical chemistry"

Khashayar Ghandi[1,2*], *Tait Du,*[1] *Maxime Lainé,*[3] *Cody Landry,*[1] *Andres Saul,*[4] *Sophie Le Caër*[3]

[1]Mt Allison University, Department of Physics, Sackville, NB E0A 3C0, Canada.

[2]Mt Allison University, Department of Chemistry and Biochemistry, Sackville, NB E0A 3C0, Canada.

[3]LIONS, NIMBE, CEA, CNRS, Université Paris Saclay, CEA Saclay, F-91191 Gif-sur-Yvette Cedex, France.

[4]Aix-Marseille University, CINaM-CNRS UMR 7325 Campus de Luminy, F-13288 Marseille Cedex 9, France.

1. Supplementary Figures

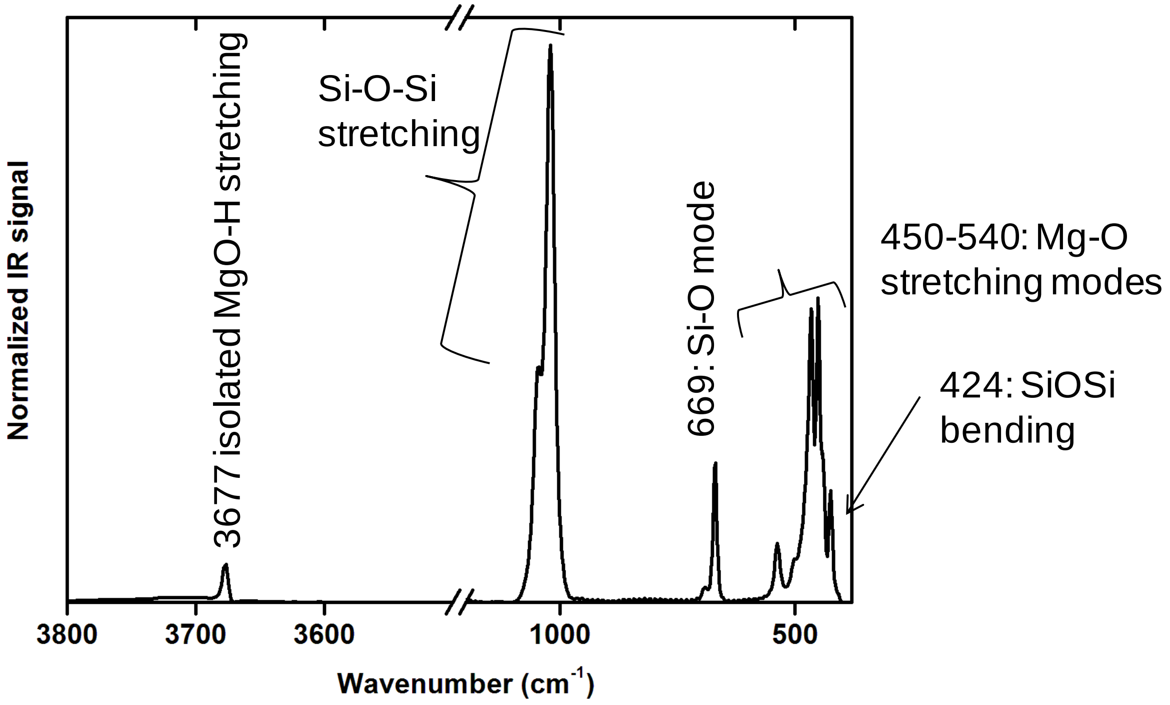

Supplementary Figure 1. IR spectrum of synthetic talc.

No water molecules were found in the sample as evidenced by the absence of the water bending mode around 1640 cm^{-1} and by the absence of a broad O-H stretching band (3000-4000 cm^{-1}). This is also consistent with TGA measurements (Supplementary Figure 2). The assignment of the different infrared bands is directly displayed in the Figure.[1]

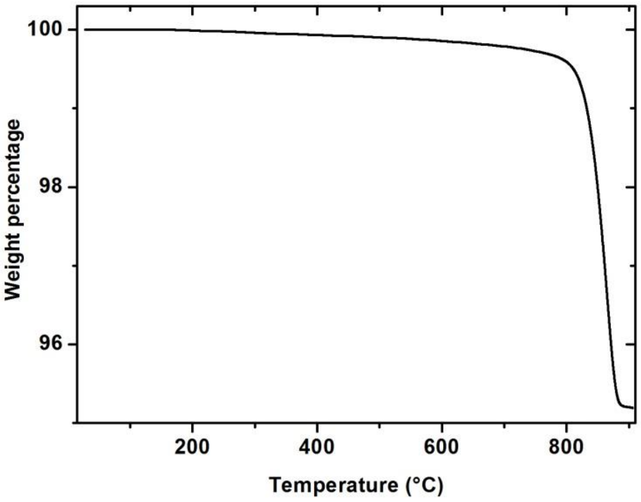

Supplementary Figure 2. TGA of synthetic talc.

Water is not detected (no weight loss around 100°C). Due to the non-swelling character of talc, the TGA is not modified by the relative humidity (not shown here).

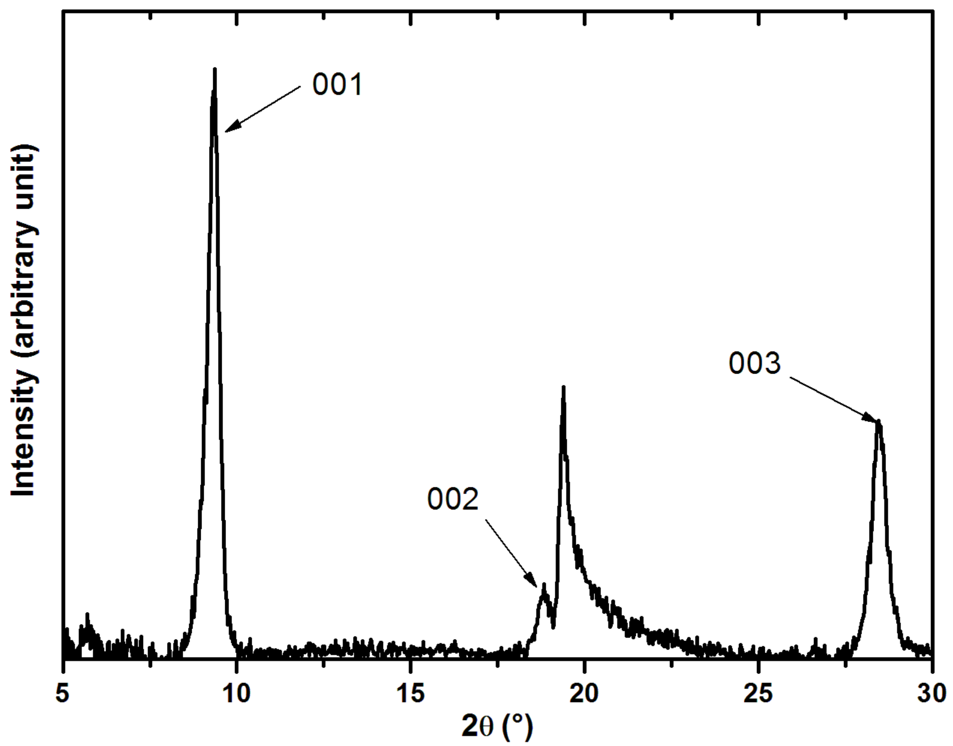

Supplementary Figure 3. XRD pattern of synthetic talc.

The d_{001} value obtained for talc is 9.43 ± 0.02 Å which proves that there is no water layer in the interlayer space.[2] The thickness of TOT being 6.5 Å, the thickness of the interlayer space is then around 2.9-3 Å.

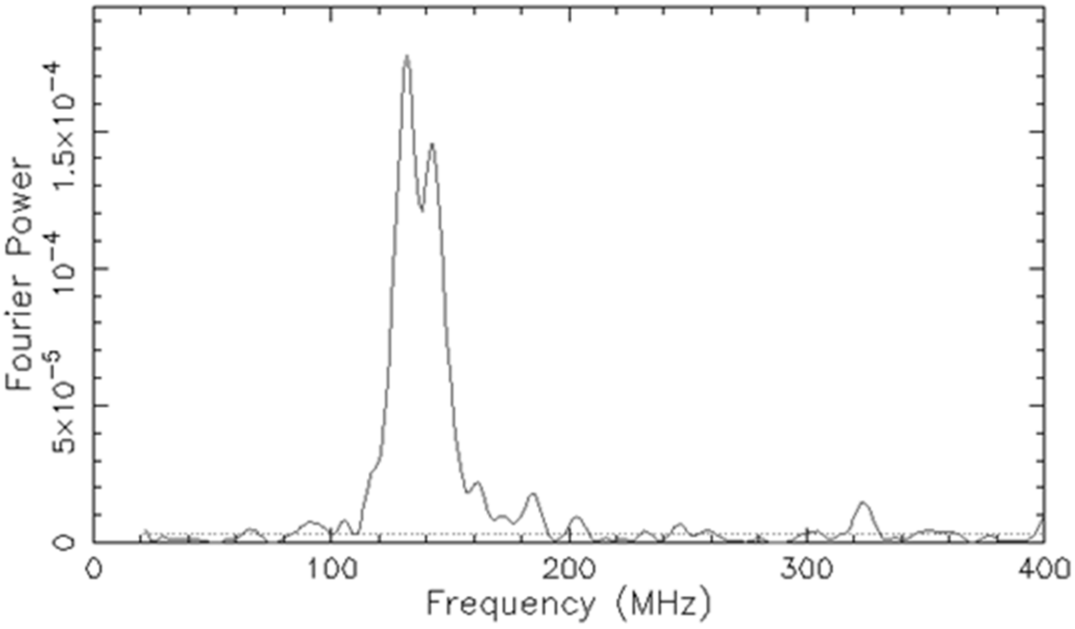

Supplementary Figure 4. Fourier power spectra of synthetic talc at 2.9 K in a transverse magnetic field of 98 G. The dotted line corresponds to the noise level.

Based on the calculations of the Breit-Rabi diagram, using the 3D Hamiltonian for hyperfine and Zeeman terms at 98 G for Mu with the HFCC close to its vacuum level, we expect two frequencies at almost 132.4 and 140.9 MHz with an amplitude close to each other (but not the same). However, we observed the peaks at 133.2 and 140.1 MHz that can be associated to Mu with HFCC of 5500 MHz. The one at higher frequency would have smaller amplitude if the Mu formation were delayed and because of instrumentation errors, namely the low time resolution. Here the Fourier power of the higher frequency is 80 percent of the lower frequency (Fourier amplitude close to 90 percent). In this case, the observed ratio of the amplitudes could be within the instrumentation factor (due to time resolution). This can indeed be observed even for prompt Mu formation (e.g. figure 4 in J. Phys. Chem. A 2004, 108, 11613-11625).

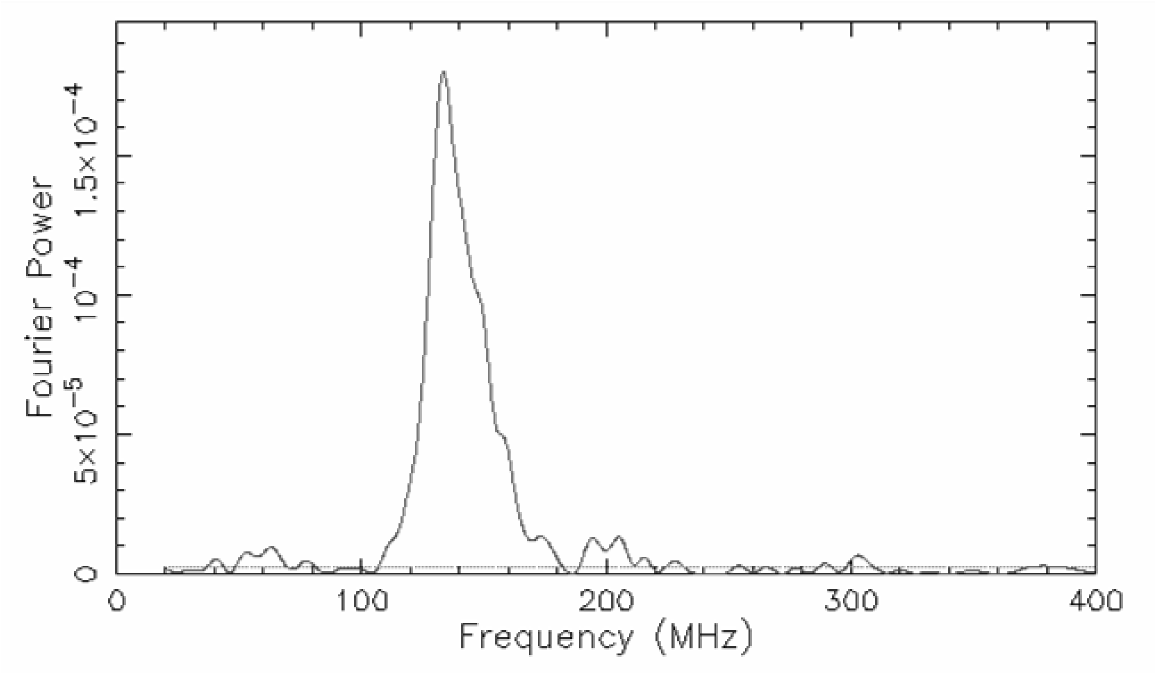

Supplementary Figure 5. Fourier power spectra of synthetic talc at 25 K in a transverse magnetic field of 97 G. The dotted line corresponds to the noise level.

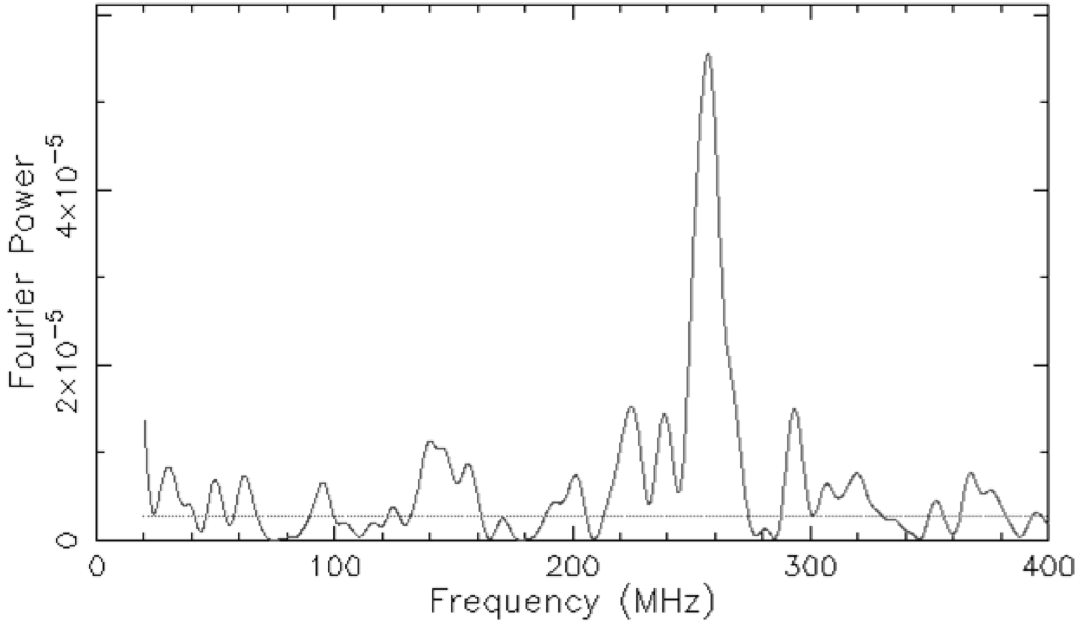

Supplementary Figure 6. Fourier power spectra of synthetic talc at 25 K in a transverse magnetic field of 196 G. The dotted line corresponds to the noise level.

The v12 is at 259.5 MHz that gives to a HFCC of 5500 MHz. Assuming a precursor with a HFCC around 190 MHz and rate of transformation to Mu around 10^8 /s the ratio of the amplitude at v23 to v12 is around 10 percent which makes the ratio of the fourier power close to 1 percent and therefore close to the noise level (explained in the text of the paper).

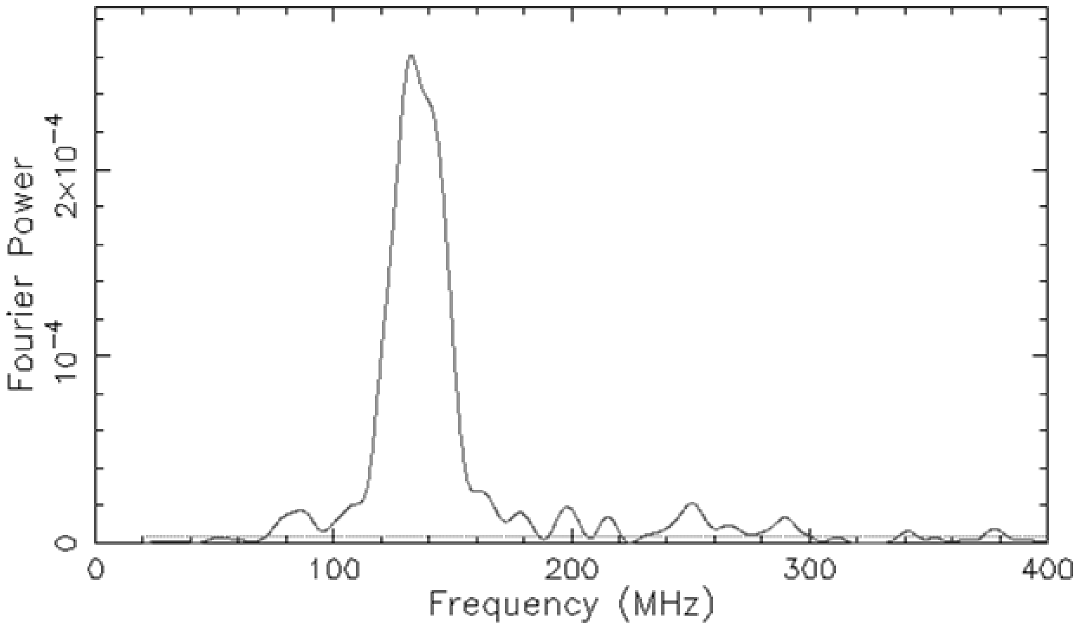

Supplementary Figure 7. Fourier power spectra of synthetic talc at 50 K in a transverse magnetic field of 98 G. The dotted line corresponds to the noise level.

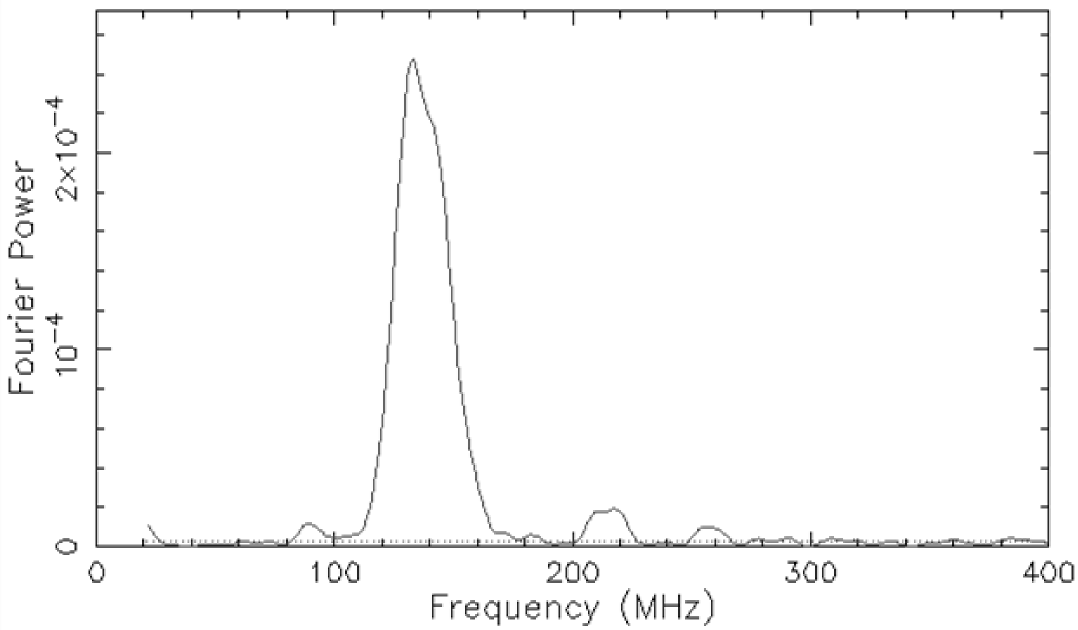

Supplementary Figure 8. Fourier power spectra of synthetic talc at 150 K in a transverse magnetic field of 98 G. The dotted line corresponds to the noise level.

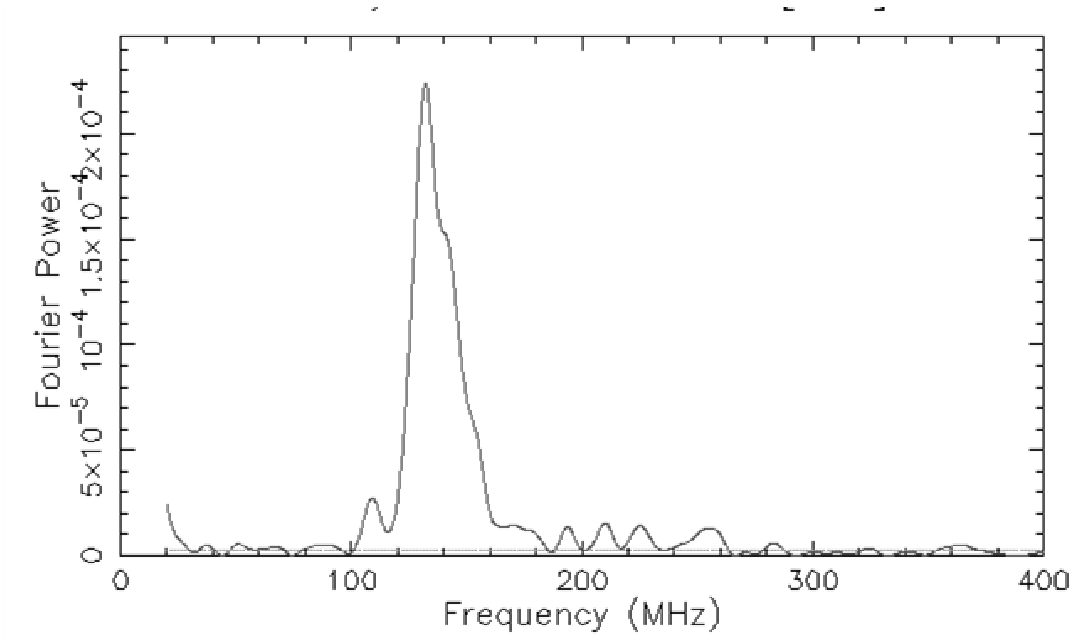

Supplementary Figure 9. Fourier power spectra of synthetic talc at 250 K in a transverse magnetic field of 98 G. The dotted line corresponds to the noise level.

2. Supplementary References

1 Farmer, V. C. The infra-red spectra of talc, saponite, and hectorite. *Mineralogical Magazine* **31**, 829-845 (1958).

2 *Handbook of Clay Science*. Vol. 5 (Elsevier, 2013).
